# Trouble in Arkansas Medical Association

**Published:** 1874-12

**Authors:** 


With the issue of our January number we will be required, under the new postal law, to prepay the postage upon the Journal to all of our subscribers. We design complimenting our readers with this prepayment of postage without any extra charge to them; and therefore trust that all will see the necessity of putting us in funds for this purpose by prompt payment of subscriptions. This is our *first* call for money through the columns of the Journal, and we hope will be the last one.

Robert Battey, M.D.,

*Business Manager.*

